# "Saving" Half-A-Million

**Published:** 1921-05-28

**Authors:** 


### "Saving" Half-a-Million.

We are glad to see that very general protests are being made against the proposal of the L.C.C. Education Committee to abandon the day continuation schools for boys and girls above the age of fifteen years. Apart from the doubtful legality of this intention—a point that we hope will not be overlooked by the Board of Education—the proposal implies a serious step backward. The London Authority had a great opportunity in the continuation school scheme to set an example to the rest of the country in the care of the mental, moral, and physical well-being of children in that dangerous period—the school-leaving age. Hundreds of local authorities were hoping to profit by the experience gained in London through this great experiment, which Sir George Newman (from the health point of view) and other equally sound experts (from the educational point of view) regarded as the most promising task ever undertaken in this country for completing the gaps in our system of education. Now, in order to save £500,000, the London Committee proposes to throw on to the streets, at the age of fifteen, many thousands of children who would otherwise have been properly cared for in body and mind, and given a sound vocational training. The arguments for the day continuation schools, said the Education Minister a few days ago, are "perfectly overwhelming." "A system of education," he added, "which spends millions of money on the education of children between the ages of five and fourteen and then does little or nothing for them during these far more important later years of a child's life is not one which will best stand critical examination." The L.C.C. Committee, having put its hand to the plough, is turning back. But in saving £500,000 it will be losing for the nation an asset which cannot be computed in terms of money.

